# Ventricular Dyssynchrony: 12-month Evaluation In Ischemic Versus Nonischemic CRT Patients

**Published:** 2009-01-07

**Authors:** Carlo Peraldo, Paolo Azzolini, Sabrina Matera, Donatella Nistri, Stefano Bianchi, Fabrizio Sgreccia, Sergio Valsecchi, Mario Davinelli, Andrea Puglisi

**Affiliations:** 1 Ospedale Fatebenefratelli - S. Giovanni Calibita, Rome, Italy; 2 Medtronic Italy, Rome, Italy

**Keywords:** Heart Failure, Resynchronization, Dyssynchrony, Echocardiography, Etiology

## Abstract

**Objective:**

Few data exist about the potential differences in the dyssynchrony status of cardiac resynchronization therapy (CRT) candidates stratified by etiology of heart failure, and about the evolution of dyssynchrony at long-term follow-up. We provided a description of intra-ventricular dyssynchrony at baseline, 6 months and 12 months in ischemic and nonischemic CRT patients.

**Methods:**

Tissue Doppler Imaging was performed in 35 CRT candidates (18 ischemic, 17 nonischemic) at baseline, and at 6-month and 12-month follow-up. A group of 11 healthy subjects was considered for comparison.

**Results:**

At baseline, the standard deviation and the maximum activation delay between any 2 segments were significantly greater in ischemic (38±33ms, 94±76ms) and nonischemic (38±24ms, 96±62ms) patients versus controls (9±7ms, 22±15ms) (all p<0.05). The average time to activation for posterior and lateral wall was significantly higher in nonischemic patients, while the anterior septum activated later in ischemic patients.

At 6-month follow-up, standard deviation and maximum delay did not vary in nonischemic while decreased in ischemic group. All changes persisted at 12 months.

**Conclusions:**

No baseline differences were observed between ischemic and nonischemic patients using studied indices. At 6- and 12-month follow-up, only ischemic patients presented a significant reduction in dyssynchrony values, although in both groups CRT did not lead to a complete normalization of LV synchronism.

## Introduction

Cardiac Resynchronization Therapy (CRT) has shown to improve symptoms and prognosis of patients affected by moderate to severe heart failure (HF) with prolonged QRS complex duration [5] demonstrated that both left ventricular (LV) reverse remodeling and clinical benefits of the therapy are maintained after one year, even if they occur to a lesser degree in patients with an ischemic etiology than in patients affected by idiopathic dilated cardiomyopathy.

Several authors assessed left intraventricular dyssynchrony in HF patients to be treated with CRT using echocardiography and hypothesized that it could predict the response to the therapy [6-8], even if more recent data seem to confute this conclusion questioning the feasibility and the reproducibility of these measures [9,10].

In these studies, in most cases the population under evaluation was not stratified by the etiology of the HF. Yu et al [11] indeed demonstrated that an echocardiographic index of left ventricular dyssynchrony is predictive of the response to CRT in two separate groups of ischemic and nonischemic patients.

To our knowledge, only Van de Veire et al [12] described dyssynchrony status of HF patients stratified by etiology and QRS complex duration, and suggested that the potential differences in the area of latest mechanical activation could have practical implications in lead positioning of a CRT system.

Although the effects of CRT on clinical and instrumental indices have been widely described at follow-up, we found no descriptions of the evolution of dyssynchrony status at long-term follow-up.

Aim of our work was then to describe intra-ventricular dyssynchrony in two separate groups of ischemic and nonischemic patients scheduled for implantation of a biventricular pacemaker and to compare them with a group of control subjects. Moreover, we sought to assess the dyssynchrony status for the two groups of patients at 6 and 12 months after implantation.

## Methods

### Patients

Forty consecutive patients implanted in our hospital with a CRT or CRT-Defibrillator device (device models 8042, 7277, 7279, Medtronic, Minneapolis, MN, USA) from May 2005 to May 2006 were included in the present study. Patients were selected according to current guidelines for the CRT: 1) severe HF (New York Heart Association (NYHA) class III or IV), 2) depressed LV ejection fraction (LVEF) (≤35%), 3) QRS showing a left bundle branch block configuration with a duration ≥120 ms, 4) patient in normal sinus rhythm,  5) optimized medical therapy [13]. Patients with a recent myocardial infarction or coronary revascularization (<3 months), or scheduled revascularization were excluded. The assignment to ischemic etiology was based on clinical history of prior myocardial infarction, prior percutaneous coronary intervention, or prior coronary bypass surgery, similar to the assignment used in large CRT trials [1-4].

Furthermore, we selected a control group of 11 age- and sex-matched healthy subjects referred to the echocardiographic laboratory for the evaluation of a cardiac murmur, with normal echocardiogram, normal LV function and no history of cardiovascular disease. The study was approved by the institutional review board.

### Study procedures

Before implant a complete clinical evaluation of patients was performed to confirm the indication to CRT. Afterwards, echocardiographic analysis was performed as detailed hereinafter. All patients then underwent a CRT system implant. The transvenous LV lead was positioned in a tributary of the coronary sinus to pace the lateral or posterolateral LV wall. After a successful implant, echocardiography was used to optimize the atrio-ventricular delay in order to maximize LV filling time [14]. Inter-ventricular pacing interval was set to default value (V-V=0 ms) and remained unmodified during the study.

Patients underwent clinical and echocardiographic evaluation at 6 and at 12 months after implant for the assessment of response to CRT and of LV intra-ventricular dyssynchrony. In the study, the same operator performed all echo assessments and was blinded to the clinical evaluation. Similarly, the same cardiologist performed clinical assessment at baseline and follow-up visits and was blinded to the echocardiographic results.

Positive response to CRT was defined as a reduction of the LVESV ≥10% with respect to the value at baseline [15].

### Echocardiographic protocol

The echocardiographic examination was performed for all patients and for the control group in the left lateral decubitus position using commercially available imaging system Sequoia C512 (Siemens AG, Munich, Germany). Each measurement was averaged over three consecutive beats during sinus rhythm. LV end diastolic and end systolic volumes (LVEDV, LVESV) were estimated using the Simpson biplane equation in the apical 4- and 2-chamber views [16].

Using color-Doppler in the apical 4-chamber view, the severity of mitral regurgitation was evaluated by measurement of the ratio of the maximum mitral regurgitation jet area by color-Doppler to the area of the left atrium. A mitral regurgitation area/left atrium area ratio ≤20%, 20-40% and >40% were classified as mild or grade 1, moderate or grade 2, severe or grade 3, respectively [17].

The LV filling time was measured as the time from the beginning to the end of diastolic mitral flow. The inter-ventricular mechanical delay (IVMD) was calculated as the time difference between the onset of the QRS and the opening of the aortic and pulmonary valves.

The 95% limits of agreement for intra-observer variability of LVESV measurements were (-18 ml to 15 ml).

### Intra-ventricular dyssynchrony assessment

Tissue Doppler Imaging (TDI) pulsed wave velocity was assessed for 3 apical views (4-chamber, 2-chamber, and long axis). The sample volume was placed in the middle of the basal segments of the 6 LV walls (namely the infero-septal, antero-septal, anterior, lateral, posterior, and inferior walls). The signal-to-noise ratio was then optimized, the Nyquist limit ranged between 10 and 30 cm/s and sweep speed was set at 100 mm/s. Time to onset of systolic velocity was measured for all segments from the onset of the QRS complex to the onset of the positive component of the regional systolic velocity, and a 6-basal segmental model of LV activation was obtained [18]. The average value from 3 consecutive beats was used in the analysis. Two indices of intra-LV dyssynchrony were used in this analysis: 1) the time difference between the longest and the shortest interval among the 6 LV walls (Max Delay), and 2) the standard deviation of the 6 intervals (T_s_-SD) [11,19-21].

The 95% limits of agreement for intra-observer variability of the time to onset of systolic velocity measurements were (-11 ms to 13 ms).

### Statistical analysis

Continuous data were expressed as means ± standard deviation. Categorical data were expressed by percentages. Differences between mean data were compared by a t-test for Gaussian variables, and by Mann-Whitney or Wilcoxon non-parametric test for non-Gaussian variables, respectively for independent or paired samples. The Bonferroni correction was applied for multiple comparisons. Differences in proportions were compared by a Chi-square analysis. A p-value <0.05 was considered significant for all tests. All statistical analyses were performed using SPSS software (SPSS for Windows, version 12.0, SPSS Inc., Chicago, IL, USA).

## Results

### Study population

Forty consecutive patients were enrolled in the study. Five of these (3 with ischemic and 2 with nonischemic cardiomyopathy) died before reaching the 6 months term and were excluded from the analysis. The baseline characteristics of the remaining 35 patients are reported in Table 1.

Patients were divided into two groups according to the etiology of the disease. The two groups did not show any significant differences at baseline, in terms of clinical or echocardiographic parameters. Only IVMD showed a trend towards greater values in the nonischemic group.

Furthermore, no differences were noticed in LV lead position that was lateral or posterolateral LV wall in all patients. During follow-up the pharmacological therapy remained stable in the study population.

### CRT response assessment

At 6 months from implant NYHA functional class, QRS duration and echocardiographic parameters indices of response showed improvement in the whole population and in the 2 groups (Table 2). The IVMD also decreased significantly. Improvement was confirmed at 12 months.

Overall proportion of responders to the therapy (patients showing a reduction of the LVESV ≥10%) was 28/35 (80%) at 6 months. Considering in the nonresponder group 5 patients who died during the follow-up, the overall response rate was 28/40 (70%). In the ischemic group, 13/18 (72%) patients responded to CRT at 6 months, but at 12 months the proportion decreased to 12/18 (67%). On the contrary, 15/17 (88%) nonischemic patients (p=0.402 vs. ischemic patients) responded to CRT at 6 months, and 16/17 (94%) (p=0.088 vs. ischemic patients) at 12 months.

### Intra-ventricular dyssynchrony assessment

Table 3 shows that Max Delay and T_s_-SD at baseline were significantly different between the two groups of patients and the control group, while no differences were recorded between the groups of ischemic and nonischemic patients.

The indices did not show any significant variations 6 months after implant in the group of nonischemic patients, while they decreased in the group of ischemic patients. Indices remained significantly higher than those of the control group.

At baseline, the first LV wall to be mechanically activated was most frequently the anterior septum: in 6/18 ischemic patients and in 6/17 nonischemic patients. For the majority of ischemic patients (6/18) the last activated LV segment was the posterior wall, while for nonischemic patients was the lateral wall (in 9/17 patients).

Table 4 reports the average time to activation of the 6 LV segments. Ischemic patients showed lower average time to activation for lateral and posterior free walls with respect to nonischemic and higher values for anterior septum.

The comparison of dyssynchrony reduction, as measured by the relative decrease of Max Delay and T_s_-SD, between patients showing maximum time to activation in lateral or posterior segments with respect to the others, did not result in any significant differences, neither for the whole population nor for ischemic and nonischemic groups. All modifications persisted at 12 months term after implant.

### Association between dyssynchrony and response to CRT

In order to seek for any associations between the response to the therapy and the dyssynchrony status, we compared baseline values of dyssynchrony indices and their changes at follow-up in the two groups of responder and nonresponder patients.

Average baseline Max Delay for responders and nonresponders was 95±63 ms and 98±91 ms, while T_s_-SD was 38±28 ms and 40±34 ms. Max Delay average variation at 6 months was 29±79 ms in the group of responders and 55±87 ms in the group of nonresponders, while for T_s_-SD we found an average difference of 11±34 ms in the responders group and of 23±33 ms in the nonresponders group. However, none of these differences was found to be statistically significant.

## Discussion

Present analysis was aimed to provide a description of mechanical dyssynchrony in a population of patients candidates to CRT and stratified by etiology, both before implant and at 6 and 12 months after implant.

### CRT response

The overall study population showed marked LV dyssynchrony at baseline and manifested a substantial clinical and instrumental improvement after 6 months of CRT, in accordance with previous experiences. The majority of patients demonstrated a significant reduction in ventricular volumes and specifically a decrease of LVESV ≥ 10% that was shown to be a strong predictor of lower long-term mortality and HF events [15]. Improvement remained stable at 12-month follow-up, confirming more recent findings [5].

Both ischemic and nonischemic patients improved their LV function, although a trend was seen towards a lower proportion of patients showing LV reverse remodeling in the ischemic group, in accordance with the Miracle Study results [5].

### LV dyssynchrony

The two indices of LV dyssynchrony used in this analysis presented clearly abnormal values in HF patients with respect to those of the control group. Moreover, no significant differences were found in global mechanical dyssynchrony between ischemic and nonischemic patients, as previously reported by Van de Veire et al [12].

However, the analysis of the activation sequence of LV segments resulted in significant differences. Even if the last activated segment was most frequently the posterior wall in both groups at baseline, the average time to activation for this segment and for the lateral wall was significantly higher in nonischemic patients. On the contrary, the anterior septum activated later in ischemic patients. This suggests that, despite similar global dyssynchrony indices, patients with HF suitable for CRT may present with a different location of mechanical dyssynchrony, which primarily seems to be related to the underlying etiology.

At 6-month follow-up evaluation, both groups presented a reduction in Max Delay and T_s_-SD values that was found to be statistically significant only in ischemic patients, may be due to the limited sample size. Nonetheless, CRT did not yield to a complete normalization of LV synchronism.

Moreover, we were not able to find any association between the improvement of dyssynchrony status and baseline location of the most delayed segment. This suggests that a successful resynchronization could not only depend on positioning the lead onto the most delayed wall.

The modifications observed in all measures of dyssynchrony at 6-month persisted at 12-month follow-up, demonstrating the enduring beneficial effects of CRT in reducing ventricular dyssynchrony.

### Main findings

In this study we confirmed the capability of indices of LV dyssynchrony such as Max Delay and T_s_-SD, in highlighting the LV mechanical activation dysfunction that characterizes CRT candidates with respect to subjects with preserved LV function. However, they were not able to describe the differences in LV activation sequence characterizing the two groups, and they seemed to fail in identifying patients that most likely benefit from the therapy.

These findings seem to confirm recent evidences demonstrating that echocardiographic markers of LV dyssynchrony are not suitable for improving patient selection for CRT [9,5]. The lack of predictive power was mainly ascribed to the low reproducibility of the proposed echocardiographic indices, specifically for complex TDI-derived measures. Similarly, our results confirm that TDI measures lack sensitivity and specificity to affect clinical decisions. Therefore these measures should not be used to preclude CRT to patients fulfilling current guidelines for implantation.

To our knowledge, this is the first description of the dyssynchrony status of patients treated with CRT at long term follow-up: at 6 months from implant we observed a significant modification of LV activation pattern which was confirmed at 12 months.

We also find out that the effectiveness of resynchronization, that is, reduction in dyssynchrony indices, is not necessarily related to positive response to the therapy in terms of LV reverse remodeling.

Late recurrent LV dilation that was reported in patients with ischemic HF was ascribed to the deterioration in LV function, possibly associated to the progressive regional loss of viable myocardium that is known to occur in ischemic heart disease, rather than the loss of efficacy of resynchronization [5]. Along this line, our findings seem to demonstrate the persistence of effective resynchronization at 6 and 12 months after implant.

These findings confirm that the management of the CRT patient during follow-up should require the careful assessment of several different clinical and instrumental parameters rather than being based only on repetitive assessment of resynchronization efficacy.

### Study limitations

The present results should be interpreted within the constraints of the study limitations. This study was performed in a small patient population. Moreover, our TDI assessment protocol included the basal segment only. The observation of mid-apical segments could provide additional information. In our study we assessed LV dyssynchrony by means of pulsed wave TDI indices of activation delay and dispersion. Requiring multiple acquisitions, the data collection with pulsed wave TDI may be influenced by beat-to-beat variability. However, also several methods with color TDI require multiple acquisitions because segments of interest lie in three different view planes. In color TDI method, the time to peak of systolic velocity is usually estimated, while in pulsed TDI both time to peak and to onset have been described. Recently, it was shown that the feasibility and the accuracy of the time to onset estimation were higher with respect to time to peak [20]. Similarly, in our series we obtained acceptably low intra-observer variability.

Finally, we did not provide any data about the relation between the site of LV pacing and the resulting activation pattern.

## Figures and Tables

**Table 1:**
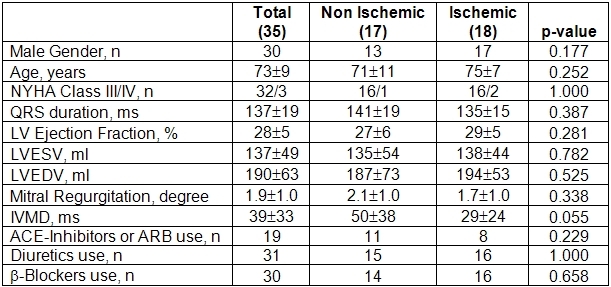
Baseline characteristics

**Table 2:**
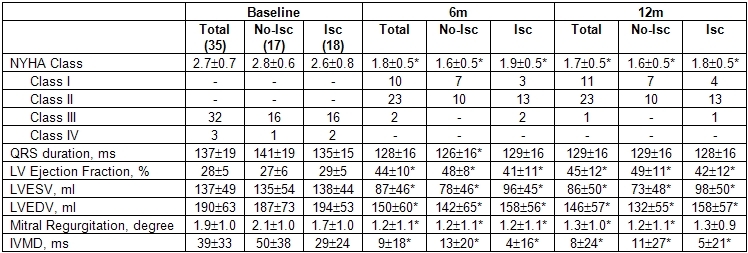
Response to CRT

* p<0.05 Vs Baseline (with Bonferroni correction)

**Table 3:**
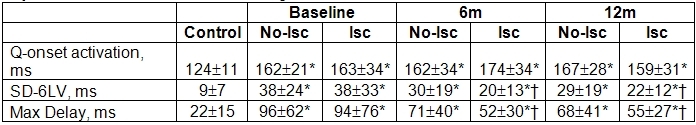
Dyssynchrony status for ischemic and nonischemic patients

* p<0.05 Vs Control; † p<0.05 Vs Baseline (with Bonferroni correction)

**Table 4:**
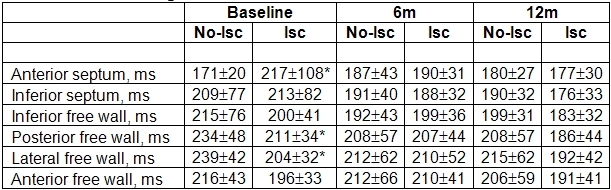
Average time to activation of the 6 LV segments

* p<0.05 Vs No-Isc (Isc: ischemic)
